# Professional Master’s Degree Students’ Perceptions on the Changes Digitalisation Imposes on Counselling in the Social and Health Care Sector

**DOI:** 10.3390/ijerph17176243

**Published:** 2020-08-27

**Authors:** Piia Silvennoinen

**Affiliations:** Unit of Digital Education and Master Programmes, Laurea University of Applied Sciences, 02650 Espoo, Finland; piia.silvennoinen@laurea.fi; Tel.: +358-46-856-7933

**Keywords:** digitalisation, online counselling, blended professionalism, social and health care

## Abstract

The study portrays the social and health care master’s students’ (*N* = 19) perceptions on the changes in work practices due to digitalisation, with a special focus on online counselling. Furthermore, the data include thoughts on the impact digitalised interaction, i.e., online counselling, has on their work and professional identities. The students studied how the motivational interviewing method combined with a solution-focused counselling approach is applicable in online settings by using simulation pedagogy. The data consisted of students’ learning diaries. Furthermore, the data were analysed using inductive content analysis. The results show that the digitalisation of work practices demands blended professionalism, which allows the professional to work both face-to-face and online with clients. In addition, the education of social and health care professionals needs to address the lack of competences and skills in digitalised work practices and provide a conceptual and practical understanding of blended professionalism in the sector.

## 1. Introduction

Since 2010, the Finnish government has initiated and undertaken the task of restructuring and reforming the Finnish health and social care system. The problems regarding the system’s functionality had become evident over the previous decades. The health and social care system reform (SOTE) aims for citizens’ equality and equal access to services, integration of services and significant cost savings through, for example, the digitalisation of numerous social and health care services and practices. The increased costs are due to the rapidly ageing population, as well as economic and geographic disparity of municipal health care and social services [1,2]. Service integration aims to both create coherent service chains and ensure services for frequent service users [3,4].

It is expected that the coordination and integration of social and health care services can lead to patient-centred care and service, and in turn increase quality and improve efficiency of the services [2]. In the future, patients and clients will have a greater freedom to choose their care and service providers. In order to assist and counsel patients and clients in the decision-making, the professionals of social and health care services must possess client-centred communication and counselling skills both in face-to-face and online settings, because a great deal of the services will be available digitally in the future [5,6]. Professionals need to be capable in communicating with patients and clients via, for example, computers, chats, videoconferences and other digital devices. In addition, professionals are in a critical role in motivating and guiding patients and clients to use eHealth and eWelfare services [7].

As studies [1,8,9] reveal, health and social care professionals lack appropriate competences and skills needed in the digitalised working life. Encompassing both knowledge of digital technology and digital skills are required to provide good patient care in health care and client service in social services [8,9]. These requirements include the associated social and communication skills, and ethical considerations of work practice digitalisation related to patients and clients [8]. The study on the integrated care competences among the workforce in the health and social care services sector in the context of the national SOTE reform reveals that the workers had the highest competence needs in advising the client on the use of digital services and guiding the client through these services [1]. Therefore, the role of regular education is essential for acquiring and maintaining the digitalisation competences in social and health care [1,8,9].

In addition, there is a turn from embodied professionalism to disembodied professionalism due to the digitalisation of work practices [10]. This in turn alters the social and health care professionalism from bodily care and contact to a direction of digitalised care and services [10]. The turn also promotes the patient and client’s self-monitoring and self-care [11].

It should be acknowledged, however, that while disembodied professionalism offers opportunities for workers’ self-management, as well as evaluation of accountability and transparency of service processes, e.g., via digitalisation, it may disregard the importance of embodied practices for the workers’ professional self-images and client relations [10]. In addition, the notion of professionalism as an embodied practice is based on the shared cultural values of the social and health care professions—it is embedded in their institutions and societies [12]. The disembodied professional practices rupture and change the structures for building and maintaining trust in the practices of the respective professions. As a consequence, trust is established on information flow, data and informatics rather than on physical and situational encounters with the clients and patients [12].

To move from provider-centred towards patient- and client-centred relationships and interaction, health care professionals must work within the conceptual world of the patient and the client [13]. One way to achieve and maintain patient- and client-centred relationships as well as communication is to acknowledge the methods for concurrent application of motivational interviewing (MI) and solution-focused counselling (SFC). These methods can form the foundation for client-centred care and service in social and health care services [13,14]. Both MI and SFC are client-centred counselling approaches for helping people to change and take the responsibility for their own actions and decisions. These approaches employ particular ways of conversing (MI) about change while cultivating and utilising client resources, i.e., strengths, abilities and intrinsic motivation (SFC) [13,14]. Thus, the client is the one who articulates the arguments for change [15,16]. The integration and congruent adoption of both MI and SFC in client-centred counselling enables multifaceted counselling with a diverse clientele [14]. As studies reveal, MI has been used in online settings to elicit change in attitudes and behaviours [17,18,19,20,21].

However, a study was conducted on the Finnish health and social services professionals’ self-evaluation of the competences in motivational interviewing and solution-focused counselling. According to the study, even though the professionals have internalised client-centred values as a premise of their work, their basic skills are partly inadequate, and their counselling competences are based on tacit knowledge [22]. In addition, the health and social care professionals lack online communication skills [8], and therefore client-centred counselling skills should be trained in social and health care degree education programmes [22].

### The Current Study

In the following section, I will portray the social and health care master’s students’ (*N* = 19) perception on the changes in work practices due to digitalisation and especially online counselling. Furthermore, the data include thoughts on the impact digitalised interaction, i.e., online counselling, has on the students’ work and professional identities. The students studied how the motivational interviewing method combined with a solution-focused counselling approach is applicable in online settings by using simulation pedagogy. The aim in the congruent adoption of both MI and SFC as the premise and foundation of client-centred counselling was to equip the students with a counselling style that has a certain starting point—the structured way of conducting counselling (MI), which simultaneously allows flexibility in counselling procedures (SFC) and functions with diverse clientele. Simulation pedagogy is a method in which teaching and learning happens through simulations of authentic cases [23]. It provides students with opportunities to practice skills in a safe environment where mistakes can be made safely without negative consequences for others [24].

The research question of the study was the following:

- What kind of effects does work practice digitalisation and especially online interaction with clients have on your work?

## 2. Materials and Methods

### 2.1. Research Setting and Participants

The participants of the study were social and health care master’s degree programme students from a university of applied sciences located in Southern Finland. The Finnish higher education system is comprised of traditional academic universities and universities of applied sciences. The master’s degree has a working life-orientated, pragmatic profile and it has been classified as an adult education degree [25]. In order to qualify for the master’s degree, applicants must have three years of work experience in the field of study, i.e., in social and/or health care sector. Studies can be carried out while working full-time [25]. Working life experience forms the context where the studies can be reflected upon, utilised and applied [25].

The students (*N* = 19) participated in a complementary course called ‘Online counselling’ during their studies in the autumn of 2018. The participant sampling of the study fulfils the criterion of purposive sampling. The sampling method allows the identification and selection of information-rich cases related to the phenomenon of interest [26]. The course comprised of three contact learning days and independent study. Contact learning days consisted of classroom lecturers and simulation demonstrations according to simulation pedagogy. As a learning assignment, the students were instructed to write a learning diary in which they reflected on their learning outcome and its relevance to their professional growth. Learning diaries are records of learners’ reflections on their learning activities represented by themselves and in their own words [27]. The students documented and reflected the following topics in their learning diaries—the possibilities and challenges of online counselling, the role of a client in online counselling, the competence requirements in online counselling and the meaning of simulation pedagogy in the learning process. The learning assignment was evaluated and graded by one of the three teachers of the course. The author (PS) of this article supervised a half-day classroom lecture for the students and she did not, for example, participate in the evaluation of students’ simulation demonstrations or learning assignments.

The informants included 16 women and three men. A total of 17 participants were health care professionals and two social service professionals. The professionals’ mean age was 37.4 years (SD = 7.27) and they had worked for between 3 to 30 years (SD = 7.48) in their field. A total of 68 percent (*n* = 13) had counselled patients or clients online once a year or never. A total of 5 percent (*n* = 1) had done online counselling a couple of times during the previous year. Furthermore, 16 percent (*n* = 3) did online counselling a couple of times in a month and 11 percent (*n* = 2) practiced online counselling with clients on daily basis.

The study was approved by the Federal Universities of Applied Sciences’ ethics committee on 1 June 2016. The students were aware of the research when signing up to the complementary course in question. They were informed that they can decline their participation in the research and still participate on the course. All the students agreed to participate in the research and signed the written agreement voluntarily. In accordance with the ethical research protocol, the students were assured that their personal data would be not be disclosed and their identities would not be revealed at any point of the research. They were informed that the participation to the research had no effect on their status as students or their course evaluation. This was verified by the fact that the researcher (PS) did not participate in the evaluation of learning demonstrations (simulations) nor learning assignments (learning diaries).

### 2.2. Data and Analysis

The research data consisted of students’ learning diaries. The qualitative research material was analysed by using inductive content analysis. Inductive content analysis is suitable for cases where there is little knowledge on the phenomenon, or when the research on the matter is fragmented. The aim of inductive content analysis is to attain a compressed and broad description of the phenomenon [28]. First, each learning diary was examined for the content that did not reflect the respective student’s own ideas and thinking, i.e., the citations of scientific literature. This content was then excluded from the analysis. Second, meaning units that in any way were connected to the research question were identified and compressed. Meaning units usually consisted of more than one sentence. Third, the compressed meaning units were then abstracted into codes, followed by the subsequent process of groupings, categorisation and finally production of the general description abstractions of the research topic through category generation [28].

## 3. Results

Analysis of the students’ learning diaries resulted in one core theme and two categories. Under each category, featured below, the content is presented by subcategories. In the results section, the word ‘professionals’ is used to refer to the master’s students of the research.

### 3.1. Towards Blended Professionalism in Digitalised Social and Health Care Work

The core theme which describes the results of the study is contextualised as blended professionalism. It highlights the professionals’ process of internalising and incorporating the digital work practices as part of their professional practices and the types of consequences it has on their professional identities. Blended professionalism entails the change digitalisation has on work, how it compresses work, and how the embodiment of work alters when the interaction with clients happens online.

#### 3.1.1. The Compression of Work

This category describes the professionals’ reflection, which stems from the societal- and structural-level explanations for the digitalisation of work practices. The professionals acknowledge that the digitalisation process of work practices is just starting and foresee how the work becomes compressed due to the needs for resource efficiency, a/synchronous work, technological solutions’ functionality and dysfunctionality, as well as security threats the technology pose.

##### Resource Efficiency

The professionals described how the demands for work efficiency and its manifestation as digital work practices has increased due to macro-level changes in society, e.g., the ageing population and geographical discrepancy in services across the country. They also understand that there is a need to curb the increased costs of social and health care services by developing easy-to-reach digitalised services, since there is a constant shortage of work force. The professionals acknowledged that digitalisation can rationalise work practices for the benefit of both the client and the professional and make services more effective.

*The organisations encounter new kinds of challenges due to the ageing population and the increase in patient numbers, so I believe that there is a pressure to develop possibilities related to online counselling for patient work.*

*I believe that online counselling could allocate more time for the tasks that require face-to-face interaction with the clients. There are many tasks that can be done online.*

##### A/synchronous Work

On the micro, i.e., personal level, the professionals describe that due to the digitalised work practices, the work performed around the computer increases. Furthermore, work can be conducted at multiple places and asynchronously, and in general the usage of digital devices and venues increases constantly. This blurs the boundary between working and private life. It also changes the rhythm of both spheres, because the idea of reachable-all-the-time becomes more and more popular.

*From a professional perspective, it is good to consider how to draw strict boundaries between the work and the spare time. Are we, the professionals, reachable 24/7? There are many drawbacks in this situation (online services), not only the fact that people are working during their spare time, but also issues concerning the security of the technology systems and legislation when working online.*

A/synchronous work will increase in the future. The interaction with clients becomes more diverse in relation to devices and venues.

*Online counselling opens new doors and possibilities for the professionals, the clients and the society. The working ways change and the appointments do not take place solely behind the closed doors, since the client can have online counselling from the professional while being at home or abroad.*

##### Functionality and Security of Digital Technology and Systems

The professionals are seriously concerned about the dysfunctionality and incompatibility issues of digital systems handling client information. The problem occurs especially in situations, where it is necessary to obtain client information from both health care and social services’ client records.

*Today all the information concerning the patient is documented to the electronic client record system of the organisation. However, the problem is that these systems are incongruent between organisations, and therefore the information from one organisation does not go forward to the other.*

The dysfunctionality of the systems, i.e., when all the needed information concerning the client is not available, disrupts work, making the processes slower, which also affects the client relationship. The security issues concerning the digital technologies in relation to identification and secure internet connection are a serious concern for the professionals. The questions of identity thefts in client encounters and ensuring that the actual client is present in the situation concern the professionals.

*In the client record systems, there are a lot of confidential medical records of the patients and because of that, there should be extra attention paid on the security issues.*

The professionals also acknowledge that a virtual presentation of an identity is not equivalent to face-to-face identification.

*In online counselling, there should be an understanding that the virtual identity of the client differs from the actual identity. It is important for the professional to understand this and keep it in mind when interacting with the client virtually.*

#### 3.1.2. The Disembodied Professionalism

This category describes the types of effects digitalisation has on the embodiment of work practices. Social and health care work is mainly based on physical, face-to-face interaction with clients. However, digitalisation alters this interaction, and aspects such as bodily care in nursing professions are replaced more and more by online interaction. Furthermore, digitalised self-monitoring and self-care of the patient will increase. These changes are seen as professionals’ views about competence, trust in client relationships and the alteration of client’s role.

##### Competence Deficit

The professionals expressed that they did not have the required competence for working with a client online. The professionals also expressed that they were eager to acquire the necessary skills. They had done very little online counselling as a part of their work, since the work practices were primarily based on the client’s physical presence.

*In the middle of the* *social and health care reform, the professional must be able to grow professionally and develop himself/herself in order to keep up with the digital leap.*

The professionals also pondered how online counselling changes and erodes their professionalism.

*When I think of online counselling … if all interaction with the clients would happen online, I would lose a great part of my expertise related to the interaction with the clients.*

They were unaware how to act with, e.g., aggressive clients in online settings.

*The video calls do not protect the social and health care professionals from inappropriate behaviour of the client. You cannot even necessarily guarantee the client’s identity in online settings. The client might endanger your personal life by photographing you while online. Face-to-face meeting ensures that if the client behaves inappropriately, you can always call the guard. How do you handle this kind of situation online?*

Also, their professional studies were conducted in the era when digitalisation had not been as advanced as today. The professionals knew how to use different digital devices from the operational point of view, but they did not have adequate awareness how the actual online interaction should be conducted. They noted that the younger clients were more at ease and even demanded online interaction, and therefore it was necessary for the professionals to gain online communication skills. In the same manner, they noted that they were worried if the older clients can choose and use the digital devices.

*It might be easier for the teenager to contact and meet the professional online rather than face-to-face, because the online* *environment is natural to them.*

*Is it possible that in the future, the only contacts that the senior citizens have are the digital ones? I believe that these (online services) function well with some patients, but not all patients have the required resources for this.*

##### Trust in Online Counselling

The professionals stated that establishing and maintaining trust in online counselling is harder and takes longer than in face-to-face interaction.

*When discussing with the client via video screen, it is important that the relationship with the client and the professional is genuinely trustworthy. The situation in which the client tells about his/her problems to the professional, is vulnerable. The task of the professional is to guarantee that the trust is established, the discussion is confidential and there are no interruptions during the session.*

Nonverbal communication and physical presence are important factors in social and health care work. In fact, the trust is established precisely through the physical presence of both parties. The online connection is also vulnerable for interruptions and disruptions. The lack of trust was portrayed in the professionals’ reflections on, for example, the validity of online counselling. They saw it less valid, and thus less trust-worthy than face-to-face counselling due to the lack of physical presence of both the client and the professional. Online interaction roused feelings of ingenuity and insecurities for, for example, how to guarantee the identity of the client.

*The idea of the world developing so much that the patient, the doctor and the health care professional all interact online and 24/7 contradicts my personal views … since face-to-face interaction is easier than online interaction. When meeting the patient face-to-face, it is much easier to consult a doctor or a colleague without making the patient to question what is actually going on with the situation.*

*On the other hand, the physical presence in the situation enables us to express empathy better (compared to online interaction), and the employee can use nonverbal signals in establishing trust between the client and the employee. It is important that the employee acknowledges the client’s genuine experiences, emotions and the fact that the online interaction is confidential.*

##### The Digitalised, Self-Sufficient Client

The digitalisation of social and health care work practices changes the role of the client. The professionals acknowledged that digitalisation not only alters the venues and ways of interaction, but also places bigger responsibility on the client to be active in choosing and gaining the adequate services. The clients must also be more self-sufficient than before. The professionals further acknowledged that the clients have become more demanding and aware of their rights.

*People are more competent and capable of advocating their rights, and they can scrutinise the data and the information gathered from* *their lives. The clients require accurate documentation on themselves and, when needed, the documentation must be revised.*

*In the future, self-help and self-care will increase as a way of maintaining good health and staying healthy. The clients’ more independent role in acquiring service will increase. In my opinion, the clients have become more active and this trend will increase in the future.*

*The deployment of online services changes health care services and increases clients’ responsibility for their own health and well-being.*

In addition, the role of the professionals is altered in the digitalisation of services, because the digitalisation affects the employees’ professional practices. The professionals stated that their role is to support the client in his/her decision-making, to motivate the clients and to recognise that the client is in charge of her/his life.

*In the counselling session, the client is not merely a target to whom the professional provides and pours all the information. The professional is not an all-encompassing authority to the client. The client is equal to the professional and the client is an expert of his/her own life. The professional can support the client in counselling. Nevertheless, the purpose of the technology is to support the client in decision-making and enable the client for the fullest self-care.*

## 4. Discussion

The main finding of the present study was that the embodied professionalism of social and health care professionals is changing into blended professionalism, which comprises both embodied and disembodied work practices due to the digitalisation of work practices. The change is fundamental, because the work of the respective professionals is, traditionally, a combination of embodied, situational and social practices of care, which are crucial to a worker’s professional identity as a skilled professional [10,12]. Blended professionalism is about conducting work both online and offline with the client [29]. In addition, the results show that blended professionalism materialises as a compression of work and disembodied professionalism. Western societies are ageing rapidly, and there is both a shortage of workforce and a need to provide more cost-effective services [2,10,29,30]. Therefore, the work-condensing process demands the necessary resource efficiency, a/synchronous work, as well as universal technological applications and solutions as a work method. These three aspects are part of a global digitalisation of work.

Due to digitalisation, work practices change into new hybrid forms of time-place embedded relationships between the client and the professional. The work is conducted via technological solutions and applications, which consequently reorganise work to be more resource efficient. However, the digitalisation of work has inherently increased the control over the clients and can thus be combined with continuum of New Public Management (NPM), managerialism and ‘assessmentality’ [10,30].

The study also revealed how digitalisation disembodies work practices and ruptures the established understanding about the social and health care professionals’ professional identities. The bodily care, e.g., in nursing professions declines and is replaced by digitalised and remote work practices [10,11,12]. Digitalisation changes the former embodied work practices from the ‘social’ to the direction of ‘informational’ [30]. In addition, the results indicate the professionals’ competence deficit regarding digitalised work practices. The result is consistent with other studies, highlighting the lack of adequate competences and skills of social and health care professionals in conducting work digitally [1,8,9].

Moreover, the issues relating to trust in the virtual interaction with the clients, the security of connections and secure storing of client records are of great importance in digitalised social and health care. Trust is a prominent component, e.g., in doctor and patient relationships, because it improves not only social and health care assessment, but also treatment outcomes and client satisfaction [31].

One of the core aims of the Finnish health and social care reform (SOTE) is to organise the services in a client-centred manner [1,2]. In a client-centred approach, the professional is a guide who enables the client to make the decisions concerning his/her well-being [5,6]. However, digitalised social and health care services increase the client’s responsibility for, e.g., self-monitoring and self-management of well-being [11]. Therefore, it is essential for the professional to acknowledge that digitalised social and health care services are based on the shared professionalism between the client and the professional. Furthermore, the clients differ in their ability to be self-sufficient [11,30].

The findings of the study are subject to some limitations. The data are rather small (*N* = 19) and they were gathered by using purposive sampling. However, the selection of respondents in qualitative research do not follow the procedures of quantitative sampling since the purpose of qualitative research is not to count opinions or people but explore the range of opinions and different representations of an issue [32]. The sample size in qualitative research is often small since the aim is to gain an in-depth understanding of a phenomenon [33]. In addition, the purposive sampling of the participants of the study followed two key considerations that guide the sampling methods in qualitative research, namely appropriateness and adequacy of the sample [34]. The participants of the study were the professionals of the social and health care sector who were interested in learning online counselling. As for the generalization of qualitative research findings, they are transferable to those people, settings, sociopolitical contexts, and times that are most like those in the focal study [34].

## 5. Conclusions

The central finding in this study was that digitalization of work practices alters embodied professionalism into blended professionalism in the social and health care sector. Therefore, it is important that the curricula of professional education and training take into account the competence requirements of blended professionalism. However, the role of the education is not to merely provide the required competences and skills, but also to provide a larger understanding of how digitalisation affects us all. As Buongiorno (2019) states, we are increasingly technologizing our bodily and cognitive abilities. It is through our body and bodily experience, i.e., senses, emotions, limbs and movements that we use and control the digital devices and tools and perform digital activities [35]. Therefore, this technologization is a process of double-embodiment, in which we extend ourselves into reality by means of digital services [35]. In turn, these devices become embedded into our bodies, blurring the lines between the organic and digital dimensions [35].
